# AU-Rich Element-Mediated mRNA Decay Can Occur Independently of the miRNA Machinery in Mouse Embryonic Fibroblasts and *Drosophila* S2-Cells

**DOI:** 10.1371/journal.pone.0028907

**Published:** 2012-01-13

**Authors:** Stephanie Helfer, Johanna Schott, Georg Stoecklin, Klaus Förstemann

**Affiliations:** 1 Gene Center, Department of Biochemistry, Ludwig-Maximilians-Universität München, München, Germany; 2 Helmholtz Junior Research Group Posttranscriptional Control of Gene Expression, German Cancer Research Center DKFZ-ZMBH Alliance, Heidelberg, Germany; French National Center for Scientific Research - Institut de Biologie Moléculaire et Cellulaire, France

## Abstract

AU-rich elements (AREs) are regulatory sequences located in the 3′ untranslated region of many short-lived mRNAs. AREs are recognized by ARE-binding proteins and cause rapid mRNA degradation. Recent reports claimed that the function of AREs may be – at least in part – relayed through the miRNA pathway. We have revisited this hypothesis using *dicer* knock-out mouse embryonic fibroblasts and cultured *Drosophila* cells. In contrast to the published results, we find no evidence for a general requirement of the miRNA pathway in the function of AREs. Endogenous *ier3* mRNA, which is known to contain a functional ARE, was degraded rapidly at indistinguishable rates in wild type and *dicer* knock-out mouse embryonic fibroblasts. In cultured *Drosophila* cells, both ARE-containing GFP reporter mRNAs and the endogenous *cecA1* mRNA were resistant to depletion of the mi/siRNA factors *dcr-1*, *dcr-2*, *ago1* and *ago2*. Furthermore, the *Drosophila* miRNA originally proposed to recognize AU-rich elements, miR-289, is not detectably expressed in flies or cultured S2 cells. Even our attempts to overexpress this miRNA from its genomic hairpin sequence failed. Thus, this sequence cannot serve as link between the miRNA and the AU-rich element mediated silencing pathways. Taken together, our studies in mammalian and *Drosophila* cells strongly argue that AREs can function independently of miRNAs.

## Introduction

The output of a gene is determined by its rate of transcription, the post-transcriptional processing and stability of the mRNA, its translation rate and the post-translational control of protein activity and stability. Despite the fact that cellular mRNAs share a common set of important structural features like the 5′-cap and poly-A tail, large variations in mRNA half-life are observed, e.g. spanning from less than one hour to >24 h in mouse ES cell lines [1]. The degradation rate of mRNAs is determined by specific regulatory sequences, for which the family of AU-rich elements (AREs) is a well studied example. They were discovered in the 3′-untranslated region (3′-UTR) of unstable mRNAs coding for cytokines [2]. When transposed into an otherwise stable mRNA, AREs cause the mRNA to be deadenylated and degraded rapidly. Based on sequence differences and deadenylation kinetics, several classes of AREs have been defined [3]. Presumably, different classes of AREs recruit distinct sets of RNA-binding proteins, resulting in differential regulation. For example, Tristetraprolin (TTP) binds to class II AREs that typically occur in cytokine mRNAs and causes rapid ARE-mediated mRNA decay [4]. The destabilizing activity of TTP, however, is not constitutive: It can be temporally masked through phosphorylation of TTP by the mitogen-activated protein kinase-activated protein kinase 2 [5]. This additional level of control helps to generate a transient peak of cytokine expression (reviewed in [6]). While in one case the recognition of an ARE by a paralog of TTP has been elucidated via crystallography [7], it is not clear how binding affinity and specificity are achieved in other cases. Because functional AREs normally contain clustered repeats of the characteristic AUUUA motif, cooperative binding of several factors in close proximity has been proposed [3].

Small non-coding RNAs such as microRNAs (miRNAs) and short interfering RNAs (siRNAs) also lead to the destabilization and translational repression of cognate mRNAs. They are bound by proteins of the Argonaute family (Ago) and confer target mRNA specificity to the RNA-induced silencing complex (RISC). Based on data obtained in *Drosophila* and human cell cultures, Jing *et al.* [8] proposed that miRNAs with appropriate sequence complementarity can recruit RISC to the ARE and thus mediate part of the repressive effect. In another report, Vasudevan and Steitz demonstrated that recognition of an ARE by miRNA-loaded RISC, in association with the Fragile-X mental retardation-related protein 1 (FXR-1), activates mRNA translation in quiescent cells [9], [10]. This is surprising given that AREs and miRNAs are both generally regarded as negative regulators. Cross-talk between the miRNA and the ARE pathway has also been observed in liver cells. When grown under normal conditions, CAT-1 mRNA is targeted by miR-122, leading to its re-localization into P-bodies and translational suppression. Upon amino acid starvation, the ARE-binding HuR protein is translocated into the cytoplasm and recruited to an ARE in the CAT-1 mRNA, downstream of the miR-122 binding site. Recruitment of HuR causes miR-122 to dissociate, whereby CAT-1 mRNA is released from P-bodies and resumes translation [11].

While most work on ARE-mediated regulation has been performed in mammalian systems, it has become clear that *Drosophila melanogaster* is not only capable of recognizing and degrading mRNAs containing mammalian AREs [8], [12], [13], but also uses this system to control endogenous transcripts, e.g. the mRNA coding for the antimicrobial peptide cecropin (cec)A1 [14]. Moreover, the TTP-homolog *tis-11* was found to be essential for this regulation in *Drosophila* [8], [12] and overexpression of *tis-11* results in a further increase of the mRNA degradation rate. We revisited the proposal by Jing *et al.* [8] that miRNAs are required for degradation of ARE-containing mRNAs. To this end, we examined AU-rich element mediated decay in mouse embryonic fibroblasts lacking the essential miRNA biogenesis factor *dicer*, and found that the half-life of endogenously expressed *ier3* mRNA, an ARE-containing transcript, is unchanged. In cultured *Drosophila* S2 cells we observed that ARE-containing reporter mRNAs as well as endogenous cecA1 mRNA are repressed in a *tis-11* dependent manner. Again, this repression did not depend on components of the miRNA or siRNA pathways. Direct inhibition or overexpression of miRNAs with potential sequence-complementarity to the ARE did not change reporter gene expression. Finally, we demonstrate that the putative precursor of *Drosophila* miR-289 – the miRNA that was originally found responsible for ARE recognition in *Drosophila* - does not give rise to a mature miRNA. We therefore conclude that in mouse embryonic fibroblasts and *Drosophila* S2 cells, ARE-mediated post-transcriptional control is independent of the mi/siRNA pathways.

## Results and Discussion

### Efficient ARE-mRNA decay in Dicer-deficient mouse embryonic fibroblasts

**Figure 1:**
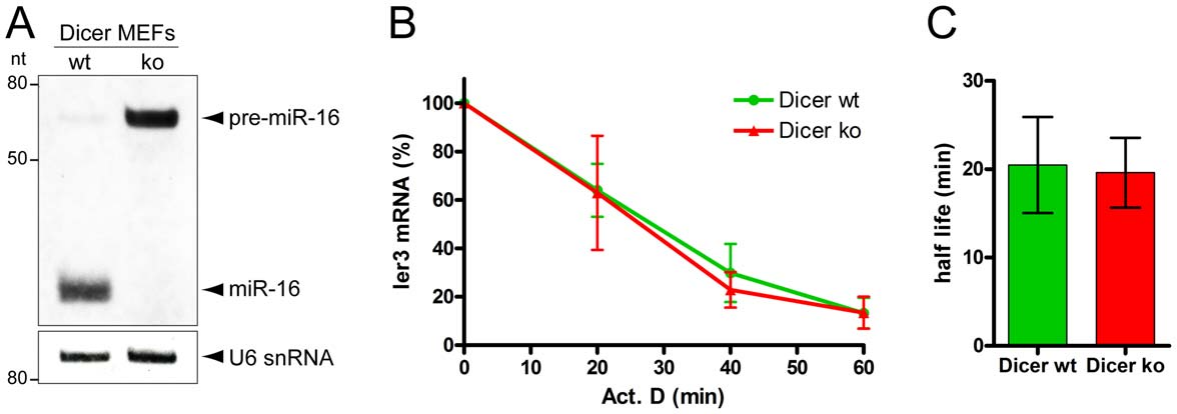
ARE-mediated mRNA decay is independent of Dicer in MEFs. A) Total RNA was extracted from wt and *dicer* ko MEFs, and the expression of miR-16 was examined by Northern blot analysis. *dicer* ko MEFs show an accumulation of the precursor pre-miR-16, whereas mature miR-16 is detected only in the wt MEFs. U6 snRNA serves as a loading control. B) Degradation of the ARE-containing *ier3* mRNA was examined in wt and *dicer* ko MEFs. Cells were treated with 5 µg/ml Actinomycin D and RNA was isolated at the indicated time points. Then, *ier3* mRNA levels were measured by quantitative real-time PCR and normalized to nucleolin mRNA levels. Shown are average values ± SD from three biological repeats. C) *ier3* mRNA half-lives were calculated from the decay curves, and plotted as average values ± SD in the graph on the right side.

In mammalian cells, mRNAs containing class II-type AREs are rapidly degraded through binding to proteins of the TTP family. In a previous report, TTP function was suggested to depend on the microRNA miR-16 [8]. In a first approach to assess the contribution of miR-16, and miRNAs in general, to the decay activity of TTP, we used Dicer-deficient mouse embryonic fibroblasts (MEFs) [15]. These MEFs fail to produce mature miR-16 and show accumulation of the precursor pre-miR-16 (Fig. 1A). In contrast, wt MEFs show prominent expression of mature miR-16.

The mRNA encoding *ier3* was identified as a target of TTP in TTP knock-out (ko) MEFs, where it is stabilized compared to wild type (wt) cells [16]. We measured decay of endogenous *ier3* mRNA in both wt and *dicer* ko MEFs, and found that the mRNA degrades at indistinguishable rates in both cell lines (Fig. 1B and C). This result shows that neither Dicer nor miR-16 are required for *ier3* mRNA decay, strongly suggesting that AREs do not genereally depend on the miRNA pathway to trigger mRNA.

### Artificial and authentic mammalian AREs repress GFP reporters in *Drosophila* cells

**Figure 2:**
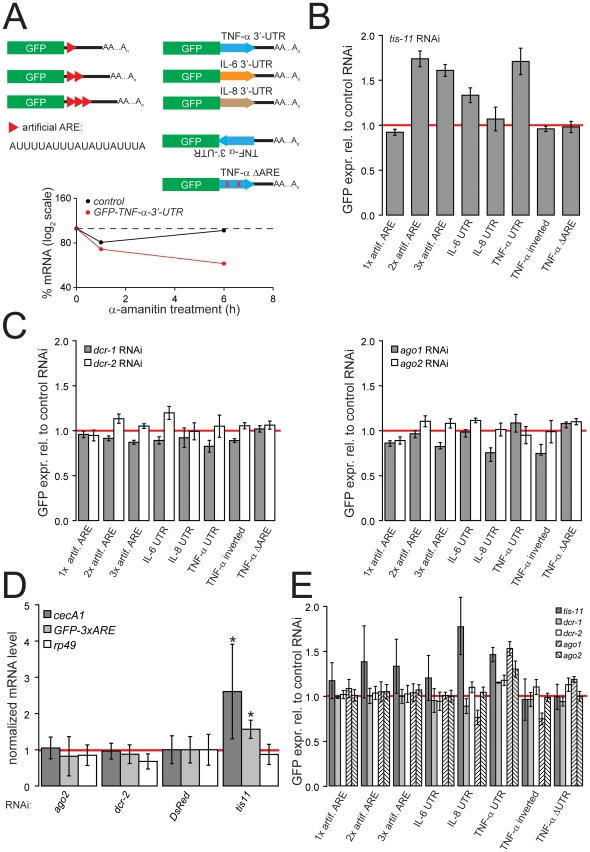
ARE mediated repression is independent of the mi/siRNA pathway in *Drosophila*. A) Schematic representation of the reporter constructs employed in our analysis; the sequence of one artificial ARE element is indicated 5′ to 3′ (modeled after the interferon-γ element). Bottom panel: Analysis of reporter-mRNA decay. Transiently transfected *Drosophila* S2-cells were treated with α-amanitin at 5 µ g/ml for 0, 1 and 6 hours. Introduction of the TNF-α-3′UTR sequence destabilizes the reporter mRNA. B) Response of the GFP reporters (stable, polyclonal cell lines) to depletion of the canonical AU-rich element binding factor *tis-11*. GFP fluorescence was measured by flow cytometry and normalized to control knock-down (dsRNA directed against DsRed); values are mean ± SD (n = 3). The changes were significant for 2xARE, 3xARE, IL-6 3′-UTR and TNF-α 3′-UTR when compared to the TNF-αΔARE control (*p*<0.05, student's t-test, n = 4), C) left panel: response of the reporters (same as in B) to depletion of *dcr-1* or *dcr-2*; right panel: response of the reporters (same as in B) to depletion of *ago1* or *ago2*. D) Analysis of mRNA steady-state level changes upon depletion of *tis-11*, *ago2* and *dcr-2*. Quantitative RT-PCR analysis was performed for the indicated mRNAs (*cecA1* = endogenous ARE target, GFP-3xARE = stable ARE reporter, *rp49* = ribosomal protein mRNA), normalized to the transcript levels of *gapdh*, and changes were calculated relative to control RNAi directed against DsRed. The asterisk (*) indicates a significant change relative to control RNAi (*p*<0.03, Wilcoxon's rank sum test, n = 6). E) Experiment analogous to B) and C) using transient transfection of the reporter constructs rather than stably expressing cells.

To validate and extend the above observation to a different system, we inserted the 3′-UTR sequences of mammalian TNF-α, IL-6 and IL-8 mRNAs (identical to the ones analyzed in [8]) downstream of the GFP coding sequence in a *Drosophila* expression vector [17]. In addition, we generated GFP reporter constructs containing one, two or three repeats of an artificial AU-rich sequence resembling the ARE of *interferon*-γ mRNA in their 3′UTRs [18] (Fig. 2A). This ARE belongs to a different subclass that has not previously been tested for its function in *Drosophila* [19]. Plasmids with a deletion of the ARE-sequences within the TNF-α 3′-UTR or with the TNF-α ARE inserted in the opposite orientation served as a control. Our reporters are analogous to GFP-reporters developed for assaying AU-rich element mediated decay in mammalian cells [20], [21]. The primary read-out is GFP fluorescence, which can be measured by flow cytometry. We validated that the TNF-α-3′-UTR sequence destabilizes the reporter mRNA by measuring the decay after transcriptional inhibition through α-amanitin (Fig. 2A, bottom panel). Consistent with a decreased half-life, the steady-state levels of GFP-TNF-α-3′-UTR mRNA were about fourfold lower than the control mRNA before α-amanitin treatment (data not shown). We selected stable cells to avoid variations due to differences in transfection efficiency that are frequently observed with transient transfection assays. Subsequently, the *Drosophila* TTP-homolog *tis-11* was knocked down by exposing the cell lines to *tis-11*-specific dsRNA. The GFP signal was normalized to a non-specific control (dsRNA directed against DsRed). We observed that the GFP reporters containing two or three repeats of the artificial ARE sequence as well as the IL-6 and TNF-α 3′-UTRs were de-repressed upon knock-down of *tis-11*, indicating that the ARE pathway represses these mRNAs (Fig. 2B).

A discrepancy between our data and the publication by Jing *et al.* (2005) is that the IL-8 3′-UTR did not respond to *tis-11* knock down in our hands. One possible explanation may be that the ARE-motif from this particular sequence is less accessible in our construct. It is also possible that the constitutive, high-level expression of this 3′-UTR from our construct may sequester important cellular binding factors and thus selection for stable expression might counter-select against the more responsive cells (see below). In summary, we have created GFP-based cell culture reporters that responded to depletion of *tis-11* in four out of five cases.

### Depletion of si/miRNA factors does not lead to de-repression of ARE reporters

If a microRNA participates in recognition of AREs, then our reporters should be de-repressed upon depletion of miRNA biogenesis factors. We exposed the stably transfected S2 cells to previously validated dsRNAs targeting *dcr-1*, *dcr-2*, *ago1* and *ago2* [17], [22], [23] to induce RNAi, then measured reporter expression (Fig. 2C). We found no consistent change of GFP levels upon impairment of the small RNA biogenesis system. While in some cases a slight de-repression by depletion of *dcr-2* or *ago2* was measured, these changes neither correlated with the extent of de-repression observed after *tis-11* depletion, nor were they consistent with the reported effects of knocking down several components of the mi/siRNA system (*dcr-1*, *ago1* and *ago2*) in the experiments of Jing *et al.* [8]. In addition, we observed no change of steady-state mRNA levels for the GFP-3xARE reporter or the endogenous ARE containing *cecA1* mRNA upon depletion of *ago2* or *dcr-2* (Fig. 2D), while depletion of *tis-11* resulted in an increase of steady-state mRNA levels for both *cecA1* and GFP-3xARE (Fig. 2D). We did not observe any indication for translational repression of our reporter mRNA since de-repression upon *tis-11* knock down was 1.57-fold ±0.25 for mRNA and 1.61-fold ±0.07 for GFP fluorescence.

During the selection of stably transfected cells, only a small fraction of the original cell population is retained. It is thus possible that the properties of the selected cell population differ from the parental cells, for example resulting in a population of cells that bypasses the miRNA requirement in ARE-mediated decay. We therefore tested whether an influence of RNAi-factors on the ARE-reporters can be detected upon transient transfection of the constructs. In these experiments, we noted that prior depletion of certain cellular factors influenced the transfection efficiency (likely by inducing changes in proliferation rates). It was therefore necessary to normalize the results not only to the control RNAi treatment, but in addition to the value obtained for a control construct that did not contain an ARE. The additional normalization corrects for certain systematic variations, but introduces additional measurement noise. Upon depletion of *tis-11*, reporters carrying the 3′-UTR of TNF–αand IL-8 were de-repressed (Fig. 2E). A similar trend was observed for the reporters containing the IL-6 3′-UTR as well as one, two or three repeats of the artificial ARE. While de-repression of the IL-8 3′-UTR reporter occurred in the transient transfection assay, the same reporter did not respond to *tis-11* knock down in the stable cells (Fig. 2B). Importantly, depletion of si/miRNA pathway components did not result in a consistent de-repression of the transiently transfected reporters (Fig. 2E): Ago1, Ago2, Dcr-1 and Dcr-2 repressed the TNF–α 3′-UTR reporter (Fig. 2E), but all other constructs remained unaffected by knockdown of mi/siRNA pathway components. Thus, it is possible that miRNAs function synergistically with ARE-sequences in certain circumstances but we found no indication for a general requirement of miRNAs for AU-rich element function. In particular, there is no indication that such a component has been consistently lost during the selection of stable cells.

Our results contradict an earlier report where the same mammalian 3′-UTRs had been employed for a pilot screen with stably transfected *Drosophila* cells, then the dependency on mi/siRNA factors was validated in human HeLa cells [8]. There are several technical differences between the two studies: First, Jing *et al.* determined mRNA half-lives by qRT-PCR, whereas our analysis in *Drosophila* cells is based on reporter protein expression and steady-state mRNA levels. It is formally possible that mi/siRNA factors destabilize ARE-containing mRNAs and at the same time increase their transcription or translation rate. Indeed, translational stimulation by miRNAs has been reported in quiescent mammalian cells [9], [10]. However, such compensating effects are unlikely given that we observed the same extent of de-repression at the protein and mRNA level for our GFP-3xARE reporter.

**Figure 3:**
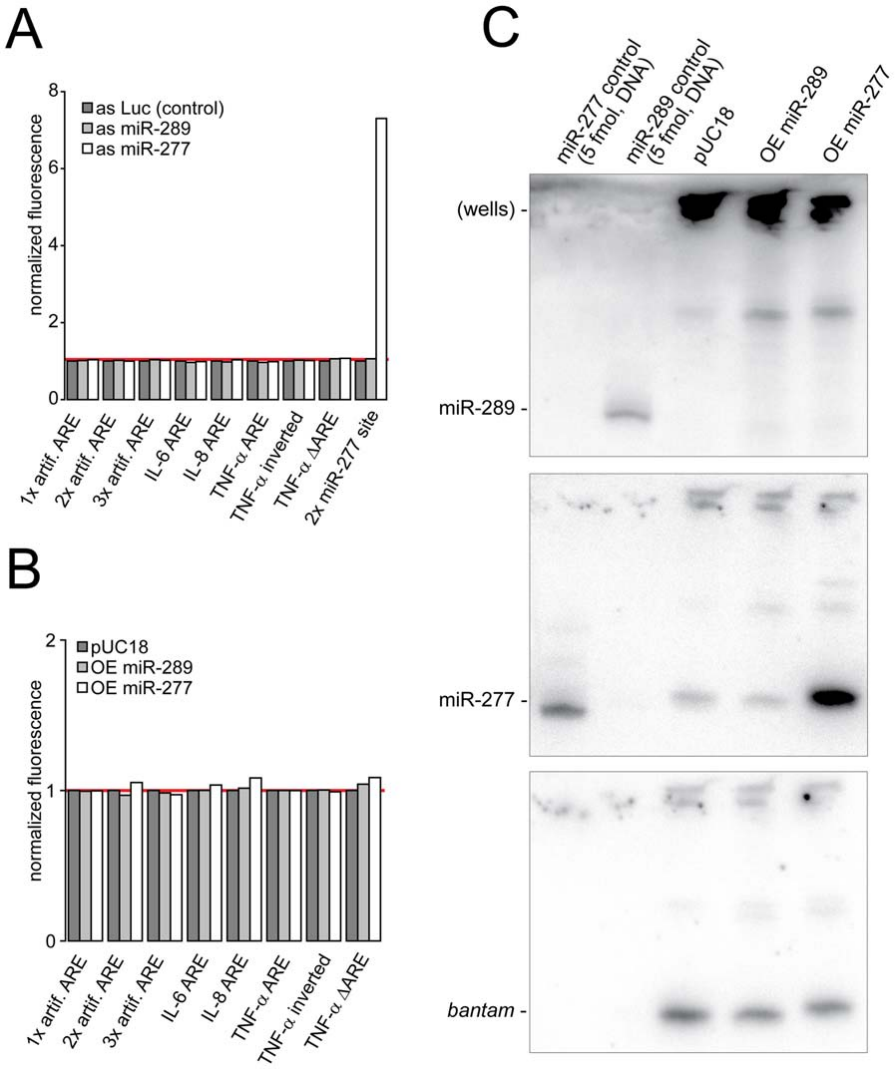
ARE mediated repression is independent of miR-289 activity. A) Response of the GFP reporters (stable, polyclonal cell lines; miR-277 reporter: stable clonal cell line) to direct inhibition of miR-277 or miR-289; an antisense oligonucleotide directed against part of the firefly luciferase coding sequence served as control for normalization; values are the mean of two experiments. B) Response of the GFP reporters (same as in A) to overexpression of miR-277 or miR-289 (transient transfection); an unrelated plasmid (pUC18) served as control for normalization; values are the mean of two experiments. C) Northern Blot to test expression of miR-289; the same membrane was stripped and hybridized with the indicated probes.

Second, the sequences in the original publication were appended to a β-globin mRNA, whereas a fusion with GFP was used in our experiments. While the choice of reporter might account for subtle differences, it is unlikely to block ARE function completely. Insertion of heterologous or artificial sequences in our experiments – as well as authentic *Drosophila* AREs [14] – into the 3′-UTR of a GFP reporter led to reduced mRNA levels. Finally, GFP reporters have also been used successfully for the analysis of ARE function in mammalian cells [20], indicating that GFP is well suited as a reporter protein for the study of AU-rich element mediated decay. Insertion of miRNA target sites into the same position of the GFP reporter results in robust repression (see Fig. 3A and [17], [22]), indicating that miRNA-loaded RISC can access this region of the reporter mRNA.

Finally, different promotors were used in the two studies to express the reporter genes, likely resulting in different steady-state reporter mRNA levels. Based on the detectable effects of *tis-11* depletion in our system, it appears that the ARE recognition system has not been saturated. Furthermore, since the same reporter system has previously been used to detect miRNA-mediated repression [17], [22], exhaustion of the small RNA response is not occurring either. Together with our results from *dicer*-deficient mouse embryonic fibroblasts (Fig. 1) this strongly argues that the small RNA silencing system is not universally required for mRNA degradation mediated by AU-rich elements.

### Direct inhibition or overexpression of miR-289 and miR-277 does not influence ARE reporters

An alternative measure to impair miRNA mediated repression is to directly inhibit specific small RNAs using modified RNA oligonucleotides of complementary sequence [24]. Jing *et al.* reported that miR-289 recognizes AREs in *Drosophila* S2 cells [8]. We therefore treated our reporter-cell lines with 2′-*O*-methyl-modified RNA oligonucleotides directed against miR-289 as well as miR-277, a second miRNA that potentially base-pairs with ARE containing sequences. Neither treatment led to an increase in ARE reporter gene expression, while a miR-277 reporter construct [17] was strongly de-repressed by the miR-277, but not the miR-289 inhibitor (Fig. 3A). In addition, overexpression of miR-289 or miR-277 did not result in any change of GFP-ARE fluorescence (Fig. 3B).

Analysis of several deep sequencing libraries published by the modEncode consortium [25] from both S2 cells and flies did not yield any sequence that corresponded fully to mature miR-289 (in contrast to 1.5×10^6^ reads matching other *Drosophila* miRNAs). Furthermore, the current version of miRBase (http://www.mirbase.org/) has included the information from 50 deep sequencing experiments for *Drosophila* small RNAs and not a single read for miR-289 is annotated. For comparison, the miR-277 precursor is annotated with 340786 reads. To ensure that this observation is not an artifact due to strong, unfavorable cloning bias, we tested whether miR-289 is expressed in our S2 cells by Northern blotting. We could neither detect mature miR-289 in control cells, whereas 5 fmol of an analogous DNA oligonucleotide were readily detected, nor in cells transfected with a vector driving expression of the putative miR-289 hairpin precursor under control of the *ubiquitin63E* promotor. In contrast to miR-289, miR-277 was readily detectable in control cells and transfection of an analogous vector expressing the miR-277 hairpin precursor resulted in strong overexpression of mature miR-277 (Fig. 3C). Taken together, we conclude that there is no evidence for the existence of mature miR-289 in *Drosophila*. It is therefore unclear how this sequence was identified as the miRNA responsible for ARE recognition in the publication by Jing *et al.* (2005). We note that miR-16, proposed by Jing *et al.* to recognize AREs in the mammalian system [8], is different from miR-369-3 identified by Vasudevan *et al.* to enhance translation of an ARE-containing reporter mRNA [10].

### Conclusion

We could not reproduce an earlier observation that the miRNA pathway is required for AU-rich element mediated control using both mouse embryonic fibroblasts and *Drosophila* Schneider cells. Furthermore, there is no evidence that miR-289 is expressed in *Drosophila*. Taken together, our findings call into question the previously published model that AREs need to be recognized by miRNAs to exert their function. Our goal is to initiate a discussion and an exchange of observations that either support or disfavor a participation of small RNAs in AU-rich element mediated regulation. It should be noted that the experiments we present certainly do not exclude the possibility that AREs and miRNA binding sites may cooperate or interfere with each other in the context of a specific mRNA.

## Methods

### Actinomycin D chase and quantitative real-time PCR

Upon the addition of 5 µg/ml Actinomycin D (AppliChem), total RNA was extracted from the cells at different times with the GeneMATRIX Universal RNA Purification Kit and 1 µg RNA was transcribed into cDNA with Transcriptor reverse transcriptase (Roche) and Oligo(dT)18 primers. After 1∶20 dilution of the cDNA, Ier3 mRNA was detected with SYBR green I (Roche) in a LightCycler 480 Real-Time PCR System using 400 nM of each primer (forward primer: 5′-GAGCGGGCCGTGGTGTC-3′, reverse primer: 5′-CTTGGCAATGTTGGGTTCCTC-3′). Nucleolin mRNA was used for normalization (forward primer: 5′-AGGGGGCAGAAATTGATGGACGAT-3′, reverse primer: 5′-TGGGTTCTGGGGCACTTTG-3′).

### RNA isolation from mouse embryonic fibroblasts and detection by Northern blot

For detection of mature miR-16 and its precursors, RNA was isolated from Dicer-deficient and wildtype MEF clones with the RNeasy Plus Mini Kit (QIAGEN). To prevent the loss of small RNAs, 100% ethanol was added to the lysate instead of 70%. About 10 µg RNA was resolved on a 15% Polyacrylamide-Urea gel and transferred for 90 min at 25 V onto a Hybond-N+ Nylon membrane (GE Healthcare) by semi-dry blotting. After UV cross-linking (2×120 mJ), the membrane was hybridized for 1 h at 37°C with a digoxigenin-labeled DNA probe specific for miR-16. The membrane was washed twice with 2× SSC/0.1% SDS for 5 min at 37°C, and twice with 0.1× SSC/0.1% SDS for 15 min at room temperature. For detection of U6 snRNA, the membrane was hybridized overnight at 42°C with a digoxigenin-labeled DNA probe. Membranes were washed twice with 2× SSC/0.1% SDS for 5 min, and twice with 0.5× SSC/0.1% SDS for 20 min at 52°C. Probes were generated with the DIG Oligonucleotide 3′-End Labeling Kit, 2nd Generation (Roche) using the following oligonucleotides: 5-CGCCAATATTTACGTGCTGCTA-3′ for miR-16 and 5′- ATCTTCTCTGTATCGTTCCAATTTTAGTAT-3′ for U6 snRNA. Alkaline phosphatase-coupled anti-digoxigenin Fab fragments and CDP-Star substrate (both Roche) were used for detection according to the manufacturer's instructions.

### Construction of *Drosophila* ARE reporter plasmids

Artificial ARE repeats were generated using the oligonucleotides 5′-GGC CAT TTT ATT TAT ATT ATT TA-3′ and 5′-CTA GTA AAT AAT ATA AAT AAA AT-3′ for one ARE; 5′-GGC CAT TTT ATT TAT ATT ATT TAA TTT TAT TTA TAT TAT TTA-3′ and 5′-CTA GTA AAT AAT ATA AAT AAA ATT AAA TAA TAT AAA TAA AAT-3′ for two AREs and 5′-GGC CAT TTT ATT TAT ATT ATT TAA TTT TAT TTA TAT TAT TTA ATT TTA TTT ATA TTA TTT A-3′ and 5′-CTA GTA AAT AAT ATA AAT AAA ATT AAA TAA TAT AAA TAA AAT TAA ATA ATA TAA ATA AAA T-3′ for three AREs with *Not*I and *Xba*I compatible overhangs. After annealing, the fragments were inserted into *Not*I and *Xba*I cut pKF63 [17].

Authentic 3′UTRs of TNF-α, TNF-α-inverted, TNF-α-ΔARE, IL-6 and IL-8 were PCR amplified from the corresponding pRMHα-3-vector (Jing, 2005) with the following oligonucleotides 5′-GCG GCC GCC CTG GCT CAC AAA TAC CAC TGA-3′ and 5′-TCT AGA TAA TTT TTG GCA GAG GGA AAA AGA TC-3′. The resulting products were inserted into the GFP-3′UTR of pKF63 via the *Not*I and *Xba*I restriction sites.

Vectors for miRNA overexpression were described previously for miR-277 [17]; for miR-289 an analogous vector was prepared by amplifying a 300 nt fragment of genomic DNA surrounding the miR-289 sequence by PCR with the oligonucleotides 5′-GAT GGA TCC TAA GCA GGC AGC ATG TCA TC-3′ and 5′-GCG GCC GCA CCA CTT CCA GCA CGT TTT T-3′. The resulting DNA fragment was then inserted into pKF63 using *Bam*HI and *Not*I restriction sites, replacing the GFP coding sequence with the miR-289 hairpin containing fragment.

### *Drosophila* Cell Culture, RNAi, transfections and FACS analysis

Dicer-deficient and wt MEFs [15] were maintained in Dulbecco's modified Eagle medium (DMEM) containing 10% fetal bovine serum (FBS superior, Biochrom AG), 2 mM L-glutamine, 100 U/ml penicillin, and 0.1 mg/ml streptomycin (all PAN Biotech) at 37°C/5% CO2. *Drosophila* Schneider 2 (S2) cells [17] were cultured at 25°C in Schneider's medium (Bio&Sell) supplemented with 10% fetal calf serum (Thermo Scientific) and Penicillin-Streptomycin mix (PAA Laboratories). Polyclonal S2 cell lines with stable expression of the artificial- and authentic 3′UTR- reporters were generated as previously described [22]. Transfection of reporter plasmids (100 ng or 500 ng) or cholesteryl-modified antisense 2′-O-methyl RNA oligonucleotides was performed as described [22]. The antisense oligonucleotides were 5′-Chol.-CAU CAC GUA CGC GGA AUA CUU CGA AAU GUC C-3′ for luciferase control, 5′-Chol-UCU AGU CGC AGG CUC CAC UUA AAU AUU UAC U-3′ for as-miR-289 and 5′-Chol-UCU UGU CGU CCA GAU AGU GCA UUU ACU-3′ for as-miR-277 (all bases were 2′-*O*-methyl modified). After transfection, cells were incubated for 4 days and then analyzed by flow cytometry in a Becton-Dickinson FACSCalibur flow cytometer.

Depletion of individual factors was performed using previously described RNAi triggers (DsRed, dcr-1, dcr-2, ago1, ago2 (Shah and Förstemann, 2008; Hartig, 2009)). To obtain an analogous construct for *tis11* the following oligonucleotides were used: 5′-TAA TAC GAC TCA CTA TAG GGA GAA TGC AAG TAC GGC GAG AAG T-3′ and 5′-TAA TAC GAC TCA CTA TAG GCC ATT CGA TGC CAA ATA TCC-3′. Cells were seeded at 1×10^6^ cells/ml (500 µl/well) and 12 µg/ml dsRNA was added to the medium. On day 4, the cells were split 1∶6 and the dsRNA treatment was repeated. The GFP fluorescence was quantified on day 8 by flow cytometry.

### Reverse transcription and quantitative RT- PCR from *Drosophila* S2 cells

After treatment with dsRNA against *DsRed*, *ago2*, *dcr-2* and *tis-11*, RNA was extracted on day 8 with TRIzol (Invitrogen) and 100 ng RNA was used for reverse transcription with Superscript II (Invitrogen), primed with a dT_18_–containing oligonucleotide (5′-ACGAATTCTTTTTTTTTTTTTTTTTT-3′).

Quantitative RT-PCR analysis was carried out with SYBR green mixes (DyNAmo-Flash SYBR Green qPCR Kit, Finnzymes) on a TOptical Thermocycler (Biometra). Primers used for amplification are: *gapdh s* 5′-CTTCTTCAGCGACACCCATT-3′ and *gapdh as* 5′- ACCGAACTCGTTGTCGTACC-3′; *rp49 s* 5′- ATCGGTTACGGATCGAACA-3′ and *rp49 as* 5′- ACAATCTCCTTGCGCTTCTT-3′; *cecA1 s* 5′- AGTCGCTCAGACCTCACTGC-3′ and *cecA1 as* 5′- CTTGTTGAGCGATTCCCAGT-3′.

### RNA isolation from *Drosophila* S2-cells and detection by Northern blot

RNA was isolated from transfected S2 cells using Trizol (Invitrogen) according to manufacturer's instructions and 3.5 µg of total RNA were used for Northern blot analysis as described previously [26]. The following probes were used for detection: 5′-TGT CGT CCA GAT AGT GCA TTT A-3′ for miR-277; 5′-AGT CGC AGG CTC CAC TTA AAT ATT TA-3′ for miR-289; 5′-CAG CTT TCA AAA TGA TCT CAC T-3′ for *bantam*.
